# Synthesis and Electrochemical Characterization of LiNi_0.5_Co_0.2_Mn_0.3_O_2_ Cathode Material by Solid-Phase Reaction

**DOI:** 10.3390/ma15113931

**Published:** 2022-05-31

**Authors:** Xinli Li, Ben Su, Wendong Xue, Junnan Zhang

**Affiliations:** 1 School of Materials Science and Engineering, University of Science and Technology Beijing, Beijing 100083, China; xinbattery@163.com (X.L.); suben111@163.com (B.S.); 2 Shandong Wina Green Power Technology Co., Ltd., Weifang 261000, China; jnzhang@winabattery.com

**Keywords:** solid-phase synthesis, carbonate, temperature, LiNi_0.5_Co_0.2_Mn_0.3_O_2_

## Abstract

In this paper, using four carbonates as raw materials, the cathode material LiNi_0.5_Co_0.2_Mn_0.3_O_2_ was prepared with the “ball milling-calcining” solid-phase synthesis method. The specific reaction process, which consists of the decomposition of the raw materials and the generation of target products, was investigated thoroughly using the TG-DSC technique. XRD, SEM and charge/discharge test methods were utilized to explore the influence of different sintering temperatures on the structure, morphology and electrochemical performance of the LiNi_0.5_Co_0.2_Mn_0.3_O_2_ cathode. The results show that 900~1000 °C is the appropriate synthesis temperature range. LiNi_0.5_Co_0.2_Mn_0.3_O_2_ synthesized at 1000 °C delivers optimal cycling stability at 0.5 C. Meanwhile, its initial discharge specific capacity and coulomb efficiency reached 167.2 mAh g^−1^ and 97.89%, respectively. In addition, the high-rate performance of the cathode sample prepared at 900 °C is particularly noteworthy. Cycling at 0.5 C, 1 C, 1.5 C and 2 C, the corresponding discharge specific capacity of the sample exhibited 148.1 mAh g^−1^, 143.1 mAh g^−1^, 140 mAh g^−1^ and 138.9 mAh g^−1^, respectively.

## 1. Introduction

With the decline in global fossil fuel reserves and the enhancement of environmental awareness, the social acceptance and demand for new energy vehicles represented by electric vehicles have continued to rise [1,2]. As one of the indispensable components of lithium-ion batteries, cathode materials have a decisive influence on the performance of lithium-ion batteries. Due to its excellent electrochemical performance, LiNi_x_Co_y_Mn_z_O_2_ with its layered structure is regarded as the most promising cathode material to replace LiCoO_2_ [3,4,5].

Currently, the main synthesis process of LiNi_x_Co_y_Mn_z_O_2_ cathode materials is the preparation of precursors using co-precipitation, which are then mixed and sintered with a lithium source to obtain the target product [6,7,8]. In this way, the mixing degree of the raw materials reaches molecular level, and the modifications of the target product in the next step are easy to achieve [9,10,11]. However, this method of co-precipitation has a high production cost and strict requirements regarding synthesis conditions, such as the temperature, atmosphere, and concentrations. Meanwhile, the reaction process inevitably generates toxic waste liquid. In addition, some researchers have prepared a LiNi_x_Co_y_Mn_z_O_2_ cathode material using sol–gel and hydrothermal methods [12,13]. These synthesis methods also have limitations, such as complicated processes and high costs. By contrast, solid-phase synthesis is a simple and effective method for preparing cathode materials. The solid-phase synthesis method consists of only two steps: mechanical mixing and high-temperature calcination. As noted in past studies, the LiNi_1/3_Co_1/3_Mn_1/3_O_2_ cathode prepared using the low-temperature solid-state reaction was compared with samples synthesized with coprecipitation. Interestingly, the former was observed to deliver a great electrochemical performance, just like that of the latter [14]. Optimizing the raw materials by using α-MnO_2_ nanorods, the LiNi_1/3_Co_1/3_Mn_1/3_O_2_ prepared with a low-temperature solid-state reaction at 900 °C shows the best electrochemical properties [15]. It is generally accepted that the microscopic morphology of Li[Ni_x_Co_y_Mn_z_]O_2_ cathode materials is closely related to the stoichiometric ratio of transition metal elements. The size of the primary particles decreases sharply with the increase in Nickel content, which leads to the expansion of the contact area between the electrodes and the electrolytes, and, in turn, it causes deterioration in the cycling stability of the cathode material [16]. A relatively large number of particles can be accessed by the “ball milling–calcining” solid-phase synthesis method, which may be beneficial to the energy density of batteries. In addition, the LiNi_0.5_Co_0.2_Mn_0.3_O_2_ prepared with solid-state synthesis not only exhibits good electrochemical performance but is also suitable for the next steps in the modification of cathode materials, such as coating and doping [17]. However, the traditional solid-phase synthesis method also has limitations, mainly manifested in the uneven element distribution of the target product caused by poor mixing uniformity of the raw materials. Optimizing the ball milling process can reduce inhomogeneity and mitigate its adverse effects on the electrochemical performance of the materials. However, in previous solid-phase synthesis studies, most researchers selected acetate/oxalate as the raw materials to obtain LiNi_x_Co_y_Mn_z_O_2_ [18], but rarely used carbonate. In terms of cost, acetate and oxalate are more expensive than carbonates.

In this paper, on the basis of optimizing the ball milling method and using carbonates as the raw materials, the LiNi_0.5_Co_0.2_Mn_0.3_O_2_ ternary cathode material was synthesized by using the “ball milling–calcining” pure solid-phase synthesis method. Thermogravimetric–differential scanning calorimetry (TG-DSC) was conducted to gain an insight into the reaction process, which includes the decomposition of MCO_3_ (M = Li, Ni, Co, Mn) and the generation of target products. Furthermore, using a variety of electrochemical testing methods, XRD, SEM, etc., the effects of calcination temperatures on the characteristics of the as-prepared LiNi_0.5_Co_0.2_Mn_0.3_O_2_ cathode materials were comprehensively studied.

## 2. Materials and Methods

### 2.1. Preparation of Cathode

Stoichiometric amounts of NiCO_3_ (98%, Macklin, Shanghai, China), CoCO_3_·xH_2_O (CP, Macklin, Shanghai, China) and MnCO_3_ (99.95%, Macklin, Shanghai, China), and 5% excess of Li_2_CO_3_ were mixed in a ball grinding jar. Using alcohol as a grinding aid and adding large, medium and small zirconia balls, the ball grinding jar was put into a planetary ball mill for mechanical mixing. The ball-to-material ratio was 2:1 and the weight ratio of large/medium/small zirconia balls was 3:4:3. The ball mill was milled in two directions at a speed of 500 rad/min for 2 h. Finally, to synthesize the LiNi_0.5_Co_0.2_Mn_0.3_O_2_ cathode material, the mixture was heated in an oxygen atmosphere for 7 h at various temperatures, namely, 800 °C, 900 °C, 1000 °C and 1100 °C.

### 2.2. Ex Site Characterizations

The thermal decomposition behaviors of the mixture in the range of room temperature to 1100 °C were investigated using TG-DSC analysis (NETZSCH STA 449 F5 Jupiter) at the heating rate of 10 K min^−1^. The crystalline phase of the as-synthesized materials was identified using powder X-ray diffraction (XRD, Ultima IV, Tokyo, Japan) with Cu Kα radiation (2θ = 10~90°). The morphology of synthesized LiNi_0.5_Co_0.2_Mn_0.3_O_2_ was observed using field emission scanning electron microscopy (FESEM Quanta TEG 450, Hillsboro, OH, USA) in a high vacuum environment.

### 2.3. Electrochemical Measurements

An amount of 80 wt% active material (as-synthesized powder), 10 wt% acetylene black (conducting additive), and 10 wt% polyvinylidene fluoride (PVDF, binder) were mixed in N-methyl-2-pyrrolidone (NMP) to prepare the slurry, which then was coated on an aluminum foil. Subsequently, the prepared electrode film was dried at 90 °C for 12 h in a vacuum environment. All the electrochemical tests were conducted using the CR2025-type coin cell (HF-Kejing, Hefei, China) in this paper. Using the as-prepared sample as the cathode, the lithium foil as the anode, 1 M LiPF_6_ dissolved in EC/DMC/DEC (1:1:1 by mass) as the electrolyte and Celgard 2400 membrane as the separator, the LiNi_0.5_Co_0.2_Mn_0.3_O_2_/Li half cells were assembled in an argon-filled glovebox. The cycling performance and high-rate charge/discharge capacity of the LiNi_0.5_Co_0.2_Mn_0.3_O_2_/Li half cells were measured using a LAND CT2001A tester (Wuhan, China) in a voltage range of 2.8~4.3 V at room temperature.

## 3. Results and Discussion

**Figure 1:**
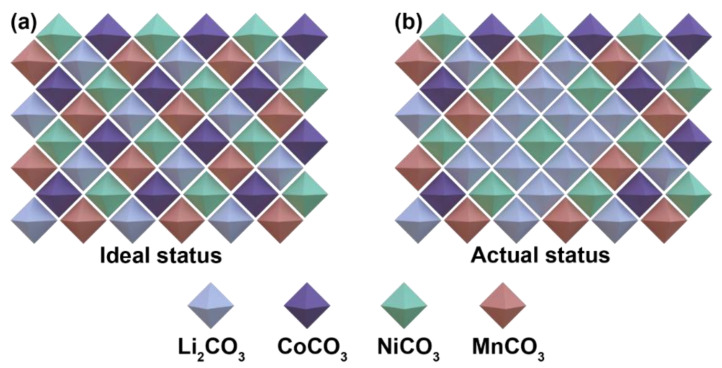
Estimation of the distribution of raw materials: (**a**) ideal status and (**b**) actual status.

In the process of synthesizing cathode materials via the traditional solid-phase synthesis method, the mixing uniformity of the raw materials is also a key factor affecting the performance of the as-synthesized materials. Under the ideal conditions of complete mixing of the raw materials, the decomposition products produced by the various carbonates decomposed at different temperatures gradually diffuse and form the target product. In fact, the preparation of LiNi_0.5_Co_0.2_Mn_0.3_O_2_ cathode materials using solid-phase synthesis involves four different transition metal salts, which are very difficult to mix completely and uniformly. In most cases, the distribution of different carbonates in the mixed raw materials may be as shown in Figure 1b.

In this situation, it is likely that one or more carbonates with a high decomposition temperature partially/completely encapsulate the carbonates with a low decomposition temperature, meaning that the wrapped material cannot gradually decompose with the increase in temperature and finally melt at a high temperature. This leads to the generation of heterogeneous phases and the loss of electrochemical performance. In this paper, we use alcohol as a grinding aid and adjust the ball milling process to enhance the mixing degree of the raw materials.

**Figure 2:**
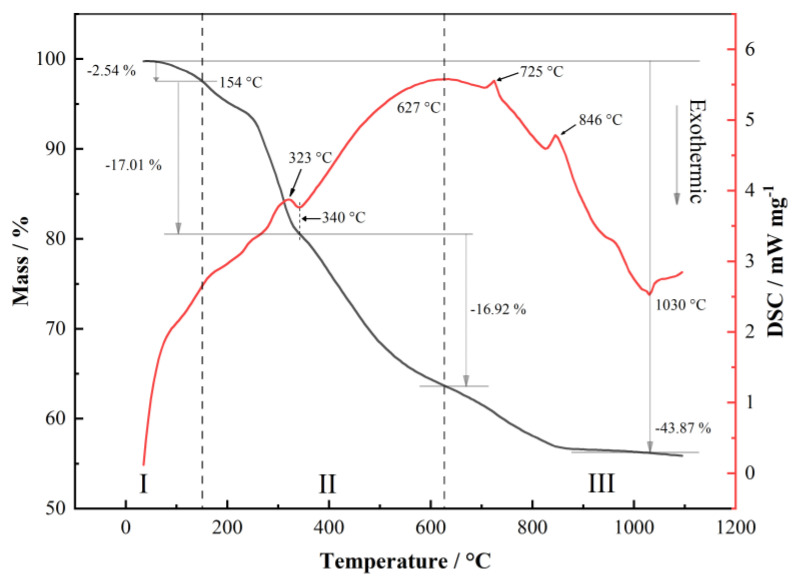
TG-DSC curves of raw materials at a heating rate of 10 K/min in air atmosphere.

Under the condition that the raw material is a mixture rather than a pure substance, the temperature at which the decomposition reaction begins and completes may change. Especially for Li_2_CO_3_, transition metal salts may play a catalytic role in the decomposition of lithium carbonate. For the carbonate raw materials involved in this study (MnCO_3_, CoCO_3_, NiCO_3_ and Li_2_CO_3_), the weight loss of the raw materials in the temperature range of 30~1100 °C is mainly related to the desorption of bound water and the gas generated by the decomposition reaction. In the differential scanning calorimetry (DSC) curve, it is generally accepted that the endothermic peak corresponds to the reaction of water loss, gas removal, the sublimation process, the decomposition process, etc., while the exothermic peak is related to the reaction of oxidation, crystallization, chemisorption, etc. In order to explore the reaction process of the carbonate raw materials at a high temperature, the compound was analyzed using thermogravimetric–differential scanning calorimetry (TG-DSC), as shown in Figure 2.

Combined with the basic principle of high-temperature solid-state reactions and the property of the materials, it is considered that the sintering process of the raw materials is mainly divided into three steps, namely, (I) dehydration, (II) the decomposition of dehydrated intermediate and (III) the generation of the target product. Step (I) occurs in the range of room temperature to 154 °C. The first mass loss of 2.54% can be attributed to the removal of adsorbed water from the carbonate mixture and the dehydration of crystallized water in CoCO_3_·xH_2_O. Step (II) occurs in the temperature range of 154~627 °C, in which the decomposition reactions of NiCO_3_, MnCO_3_ and CoCO_3_ are completed and the mass loss is caused by the decomposition of dehydrated intermediate. The endothermic response near 323 °C represents a one-time melt decomposition of NiCO_3_ that has not been decomposed [19,20,21,22]. According to the TG curve, the actual mass loss (17.01%) is higher than the theoretical calculated value of 16.77% in the temperature range of 154~340 °C due to the decomposition of other carbonates, in addition to the decomposition of NiCO_3_. The mass loss between 340 °C and 627 °C is mainly caused by the decomposition of MnCO_3_ and CoCO_3_. However, the phenomenon of mass loss still exists after 627 °C. Step (III), which occurs at 627~1030 °C, is the target product generation step. After the temperature reaches 627 °C, the DSC curve mainly shows an exothermic reaction. At this step, although the decomposition reaction of carbonates still occurs, the endothermic heat of the reaction is already much smaller than the exothermic heat of the oxidation reaction and crystallization of the target LiNi_0.5_Co_0.2_Mn_0.3_O_2_. This infers that there is still a small amount of mixing inhomogeneity, and some of the MnCO_3_ and CoCO_3_ particles melt at 725 °C at one time, thereby generating an endothermic peak. Furthermore, transition metal oxides produced by the decomposition of transition metal carbonates have a catalytic effect on the decomposition of Li_2_CO_3_, and the one-time melting decomposition of the last raw material Li_2_CO_3_ generates a final endothermic peak near 846 °C. The mixture at temperatures above 846 °C remains basically unchanged, while the DSC curve shows that there remains an exothermic process. This proves that there would be no new decomposition reactions above this temperature.

According to the TG curve, the total mass loss of the mixture from room temperature to 1030 °C is calculated to be 43.87%. After deducting the 2.54% mass loss caused by the dehydration process before 154 °C, the actual reaction mass loss is about 41.33%, which is similar to the theoretically calculated value of 40.71%. After 1030 °C, the reactions once again change from an endothermic process to an exothermic process, while the TG curve remains basically unchanged. It is believed that the high-temperature oxygen loss and sintering process of the product mainly occur above this temperature.

**Figure 3:**
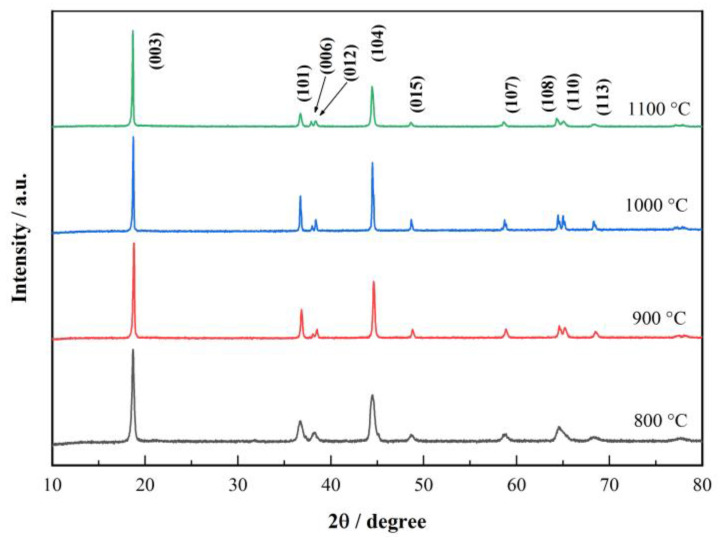
XRD spectra of LiNi_0.5_Co_0.2_Mn_0.3_O_2_ synthesized at 800 °C, 900 °C, 1000 °C and 1100 °C.

X-ray diffraction (XRD) patterns of LiNi_0.5_Co_0.2_Mn_0.3_O_2_ synthesized at various temperatures (800 °C, 900 °C, 1000 °C and 1100 °C) are shown in Figure 3.

The diffraction patterns of all samples exhibit the typical characteristics of a layered α-NaFeO_2_ structure with a group space of R$\bar{3}$m [23,24]. In addition, no peak of the impurity phase is observed in the patterns of samples synthesized at different temperatures. The split peaks of (006)/(102) and (108)/(110) are regarded as the characteristics of the layered structures [25,26]. Moreover, the ratio of I_(003)_/I_(104)_ reflects the degree of cation mixing between Ni^2+^ and Li^+^ located at the 3a and 3b sites in an ideal layered structure [27,28]. For the LiNi_0.5_Co_0.2_Mn_0.3_O_2_ prepared at 800 °C, the I_(003)_/I_(104)_ ratio is significantly less than 1.2, and the indistinguishable (108)/(110) peaks indicate that the sample is contaminated by the rock salt, which has an adverse effect on the specific capacity and stability of the material. The two split peaks of (006)/(102) and (108)/(110) on the patterns reveal the well-defined layered structure of the samples synthesized at temperatures of 900 °C and above [29]. Furthermore, the I_(003)_/I_(104)_ ratio of the samples synthesized at 900 °C and 1000 °C is around 1.2, which can be expected to show good electrochemical properties in the above samples. Combined with the analysis of the TG-DSC curve, it can be inferred that the raw materials cannot be completely converted to the target LiNi_0.5_Co_0.2_Mn_0.3_O_2_ cathode material at the calcination temperature of 800 °C. Additionally, there are some impurities generated with the emergence of the target product at 800 °C.

The particle size has a direct impact on the ease of Li^+^ deintercalation. An excessively large particle size means a longer path for Li^+^ to migrate from the interior to the surface, thereby affecting the kinetic rate of the Li^+^ deintercalation reaction and resulting in a lower rate performance [30]. However, the too small size of primary particles also increases the contact area between the electrodes and the electrolytes, which further aggravates the complex side reactions at the electrode/electrolyte interface. In other words, the too large/small size of particles damages the electrochemical performance of the materials, such as their cycling stability and high-rate charge/discharge capacity [31].

**Figure 4:**
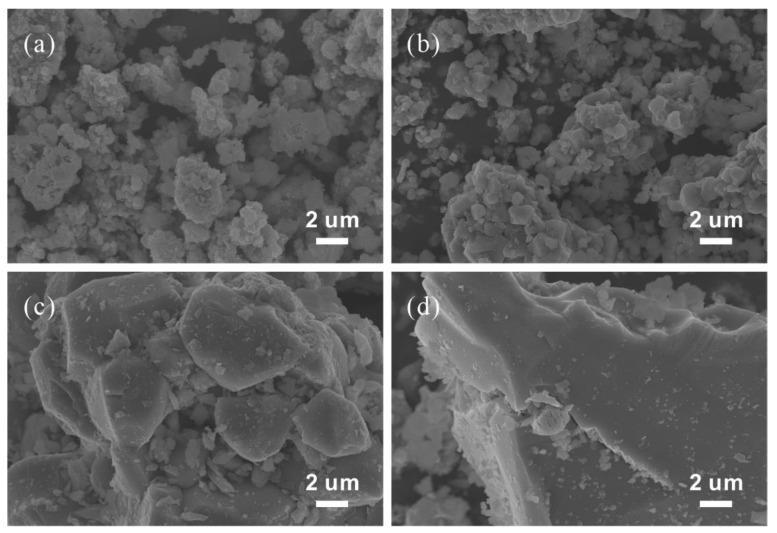
SEM images of LiNi_0.5_Co_0.2_Mn_0.3_O_2_ synthesized at different temperatures: (**a**) 800 °C, (**b**) 900 °C, (**c**) 1000 °C and (**d**) 1100 °C.

SEM images of LiNi_0.5_Co_0.2_Mn_0.3_O_2_ synthesized at different temperatures are given in Figure 4.

The particles of the samples are secondary particles composed of irregular primary particles with the obvious phenomenon of agglomeration, and they are different from the spherical LiNi_0.5_Co_0.2_Mn_0.3_O_2_ particles prepared using the co-precipitation method. Samples synthesized at the same temperature have primary particles with roughly the same size, indicating that the raw materials are uniformly mixed. In addition, it can be clearly seen from the SEM images that the size of primary particles increases with the increase in the sintering temperature (from 800 °C to 1100 °C), which is due to the rise in the calcination temperature promoting the growth of crystal grains.

**Figure 5:**
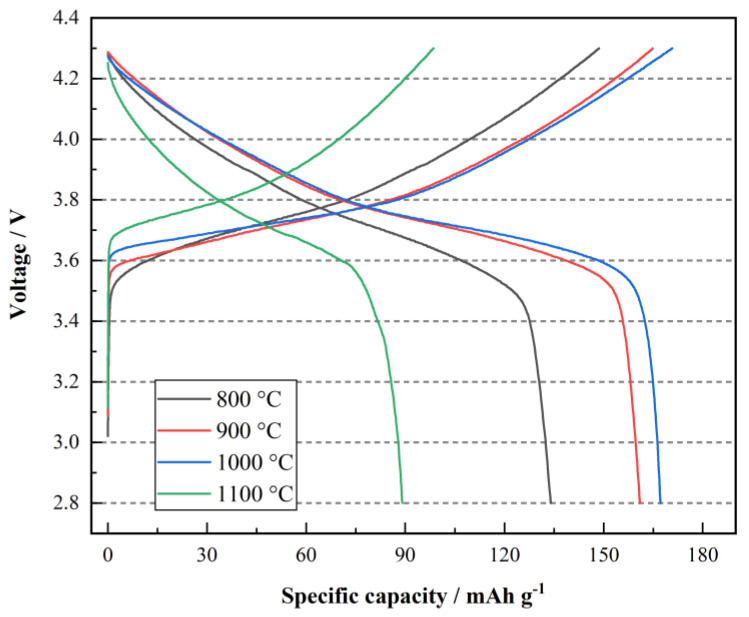
Initial charge/discharge curves of the half cells with LiNi_0.5_Co_0.2_Mn_0.3_O_2_ synthesized at 800 °C, 900 °C, 1000 °C and 1100 °C.

All the LiNi_0.5_Co_0.2_Mn_0.3_O_2_/Li half cells were precycled at 0.1 C in a voltage range of 2.8~4.3 V at room temperature for five cycles to activate the cells. The first charge/discharge curves of the four samples are shown in Figure 5.

With the sintering temperature increasing from 800 °C to 900 °C and 1000 °C, the charge/discharge specific capacity of the corresponding LiNi_0.5_Co_0.2_Mn_0.3_O_2_/Li half cells rises from 148/134 mAh g^−1^ to 164.8/161 mAh g^−1^ and 170.8/167.2 mAh g^−1^. However, the charge/discharge specific capacity of the cathode material synthesized at 1100 °C decreases sharply to 98.5/89.1 mAh g^−1^. In addition, the coulomb efficiency of the half cells with LiNi_0.5_Co_0.2_Mn_0.3_O_2_ synthesized at 900 °C and 1000 °C reaches 97.69% and 97.89%, respectively. This is also consistent with the results of XRD and SEM. Furthermore, the specific capacity of LiNi_0.5_Co_0.2_Mn_0.3_O_2_ prepared at 800 °C, 900 °C and 1000 °C is higher than that of the cathode materials synthesized using the co-precipitation method (159 mAh g^−1^). 

**Figure 6:**
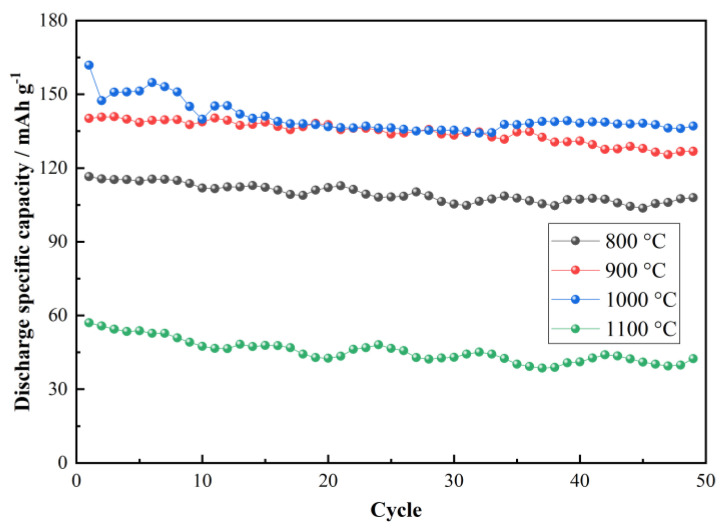
Cycling property of the half cells with LiNi_0.5_Co_0.2_Mn_0.3_O_2_ synthesized at 800 °C, 900 °C, 1000 °C and 1100 °C.

The cycling performance of LiNi_0.5_Co_0.2_Mn_0.3_O_2_/Li half cells with cathode materials prepared at different temperatures was measured at a rate of 0.5 C at room temperature (Figure 6). Upon completing cycling 50 cycles, the half cells with LiNi_0.5_Co_0.2_Mn_0.3_O_2_ synthesized at 800 °C, 900 °C, 1000 °C and 1100 °C were observed to deliver capacity retention of 80.52% (at 50th ≈ 107.9 mAh g^−1^), 78.76% (at 50th ≈ 126.8 mAh g^−1^), 81.94% (at 50th ≈ 137 mAh g^−1^) and 47.59% (at 50th ≈ 42.4 mAh g^−1^), respectively. In terms of charge/discharge specific capacity and cycling stability, LiNi_0.5_Co_0.2_Mn_0.3_O_2_ prepared at 1000 °C shows an optimal property. In addition, the poor performance of LiNi_0.5_Co_0.2_Mn_0.3_O_2_/Li half cells with the cathode prepared at 1100 °C is mainly attributed to the excessive size of the sample particles. An increase in the sintering temperature within a certain range is beneficial to the development of crystals and the electrochemical performance of the material. However, an excessively high sintering temperature may cause problems, such as material compaction and excessively coarse crystal grains, which adversely affect the electrochemical properties of the materials.

**Figure 7:**
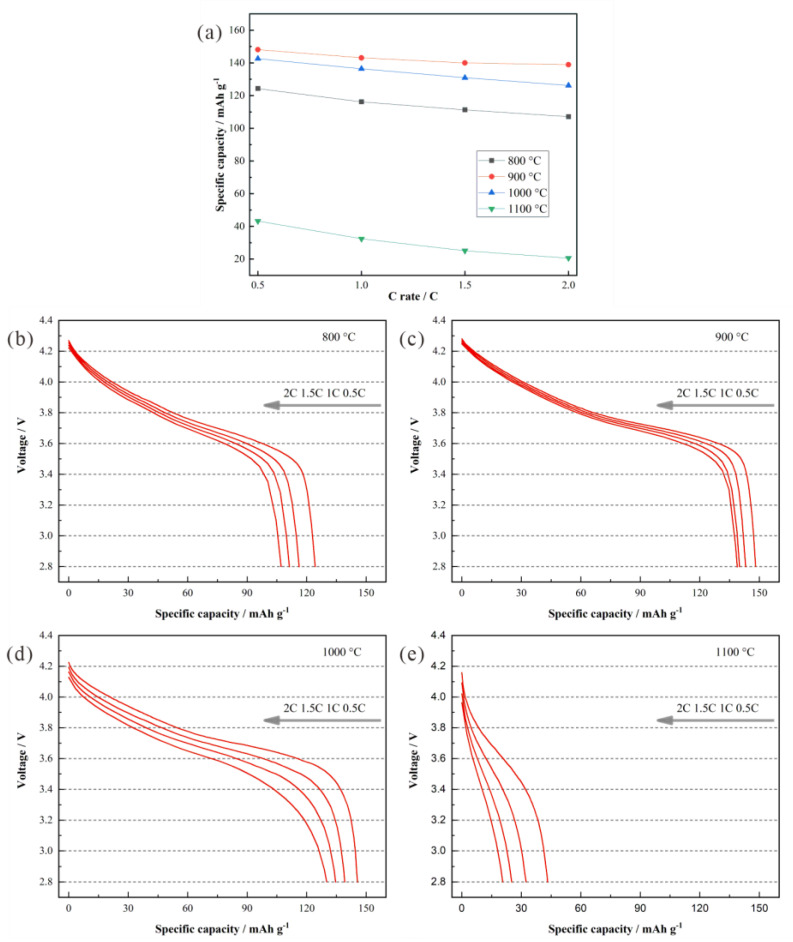
(**a**) Rate performances of the half cells with LiNi_0.5_Co_0.2_Mn_0.3_O_2_ synthesized at different temperatures and the corresponding discharge curves at the rates of (**b**) 0.5 C, (**c**) 1 C, (**d**) 1.5 C, and (**e**) 2 C.

The high-rate charge/discharge capacity is one of the most significant indicators of battery quality. Increasing the current density from 0.5 to 1 C, 1.5 C and 2 C, the rate performance of LiNi_0.5_Co_0.2_Mn_0.3_O_2_/Li half cells with cathode materials synthesized at different temperatures was examined in a voltage range of 2.8~4.3 V at room temperature (Figure 7). At the rate of 2 C, the discharge capacities of the four samples with synthesized temperatures of 800 °C, 900 °C, 1000 °C and 1100 °C are 107.1 mAh g^−1^, 138.9 mAh g^−1^, 126.2 mAh g^−1^ and 20.6 mAh g^−1^, respectively. Compared with the condition at the rate of 0.5 C, capacity retention rates reach 86.16%, 93.79%, 88.50% and 47.69%. Upon recovering the rate of 0.5 C, the specific capacity is restored to 124 mAh g^−1^, 149.8 mAh g^−1^, 140.1 mAh g^−1^ and 35 mAh g^−1^. The samples synthesized at 900 °C and 1000 °C exhibit a better rate performance and a stronger structural stability under high-rate conditions. Owing to the oversized crystal grains, lithium ions have to transport via a long pathway in solid LiNi_0.5_Co_0.2_Mn_0.3_O_2_ prepared at 1100 °C. It is widely believed that a longer pathway for lithium ions in the charge/discharge processes means a worse high-rate property of lithium-ion batteries. On the other hand, the as-prepared sample at 800 °C contains more impurities than that at 900 °C and 1000 °C, so it also delivers a poor high-rate performance. Regardless of the grain sizes or lattice structure, the samples synthesized at 900 °C and 1000 °C all exhibit good properties to support an excellent high-rate capacity. 

## 4. Conclusions

In this paper, using four carbonates as raw materials, the layered LiNi_0.5_Co_0.2_Mn_0.3_O_2_ cathode material with high specific capacity was successfully synthesized with the solid-phase synthesis method. LiNi_0.5_Co_0.2_Mn_0.3_O_2_ synthesized at 1000 °C provides an optimal cycling performance due to its high crystallinity and uniform particle size of about 2 μm. The initial discharge specific capacity and coulomb efficiency reached 167.2 mAh g^−1^ and 97.89%, respectively. On the other hand, the high-rate charge/discharge performance of LiNi_0.5_Co_0.2_Mn_0.3_O_2_ synthesized at 900 °C was particularly noteworthy, with its capacity still maintained at 93.78% at the rate of 2 C (at 0.5 C ≈ 148.1 mAh g^−1^). Restoring the current density to 0.5 C rate, its specific capacity still recovered to 149.8 mAh g^−1^. These results indicate that the LiNi_0.5_Co_0.2_Mn_0.3_O_2_ cathode material for lithium-ion batteries prepared using carbonate raw materials and the solid-phase synthesis method has broad prospects. This synthesis method not only obtain LiNi_0.5_Co_0.2_Mn_0.3_O_2_ with excellent electrochemical properties, but also greatly avoid high production costs and environmental pollution. Owing to its simplicity and efficiency, this synthetic process is well suited for large-scale commercial production.

## Data Availability

Not applicable.
